# The Arabidopsis synaptotagmin SYTA regulates the cell-to-cell movement of diverse plant viruses

**DOI:** 10.3389/fpls.2014.00584

**Published:** 2014-11-06

**Authors:** Asako Uchiyama, Harumi Shimada-Beltran, Amit Levy, Judy Y. Zheng, Parth A. Javia, Sondra G. Lazarowitz

**Affiliations:** Department of Plant Pathology and Plant-Microbe Biology, Cornell UniversityIthaca, NY, USA

**Keywords:** movement protein, cell-to-cell movement, synaptotagmin, *Turnip vein clearing virus*, *Cabbage leaf curl virus*, *Cauliflower mosaic virus*, *Turnip mosaic virus*

## Abstract

Synaptotagmins are a large gene family in animals that have been extensively characterized due to their role as calcium sensors to regulate synaptic vesicle exocytosis and endocytosis in neurons, and dense core vesicle exocytosis for hormone secretion from neuroendocrine cells. Thought to be exclusive to animals, synaptotagmins have recently been characterized in *Arabidopsis thaliana*, in which they comprise a five gene family. Using infectivity and leaf-based functional assays, we have shown that *Arabidopsis* SYTA regulates endocytosis and marks an endosomal vesicle recycling pathway to regulate movement protein-mediated trafficking of the Begomovirus *Cabbage leaf curl virus* (CaLCuV) and the Tobamovirus *Tobacco mosaic virus* (TMV) through plasmodesmata (Lewis and Lazarowitz, 2010). To determine whether SYTA has a central role in regulating the cell-to-cell trafficking of a wider range of diverse plant viruses, we extended our studies here to examine the role of SYTA in the cell-to-cell movement of additional plant viruses that employ different modes of movement, namely the Potyvirus *Turnip mosaic virus* (TuMV), the Caulimovirus *Cauliflower mosaic virus* (CaMV) and the Tobamovirus *Turnip vein clearing virus* (TVCV), which in contrast to TMV does efficiently infect Arabidopsis. We found that both TuMV and TVCV systemic infection, and the cell-to-cell trafficking of the their movement proteins, were delayed in the Arabidopsis Col-0 *syta-1* knockdown mutant. In contrast, CaMV systemic infection was not inhibited in *syta-1*. Our studies show that SYTA is a key regulator of plant virus intercellular movement, being necessary for the ability of diverse cell-to-cell movement proteins encoded by Begomoviruses (CaLCuV MP), Tobamoviruses (TVCV and TMV 30K protein) and Potyviruses (TuMV P3N-PIPO) to alter PD and thereby mediate virus cell-to-cell spread.

## Introduction

To successfully infect their hosts, plant viruses must overcome the hurdle of the plant cell wall, which is a barrier to viral exit from and entry into cells. Viruses accomplish this by encoding movement proteins to transport their genomes cell-to-cell within a leaf and on into the vascular system, through which the virus will systemically invade its host. Cell-to-cell movement occurs via a common pathway: viruses exploit plasmodesmata (PD), complex trans-wall channels that regulate the transport of solutes and key regulatory molecules between cells (Zambryski and Crawford, 2000; Oparka and Roberts, 2001; Benitez-Alfonso et al., 2010; Verchot-Lubicz et al., 2010). Movement proteins alter PD permeability to enable transport of viral genomes into adjacent cells (Waigmann et al., 2004; Ueki and Citovsky, 2011). Beyond this key role in cell-to-cell transport, movement proteins coordinate replication of viral genomes with their directed transport to PD to ensure that replication precedes virus cell-to-cell movement (Lazarowitz and Beachy, 1999). This intracellular trafficking requires interactions with the viral genome *per se*, and with ER membrane, vesicle trafficking proteins, the cytoskeleton and/or nuclear proteins, depending on the virus and its replication strategy. Some plant viruses encode additional “movement proteins” to bind the viral genome and coordinate this intracellular trafficking with the cell-to-cell movement protein. For example, Geminiviruses have DNA genomes and replicate in the nucleus. Those with two genome components (Begomoviruses), such as *Cabbage leaf curl virus* (CaLCuV) and *Squash leaf curl virus* (SqLCV) encode a movement protein that acts as a nuclear shuttle protein (NSP) to bind and transport viral genomes between the nucleus and cytoplasm. The cell-to-cell movement protein (MP) traps NSP-genome complexes in the cytoplasm and redirects them to PD and across the wall (Sanderfoot and Lazarowitz, 1996; Sanderfoot et al., 1996). In contrast, many viruses encode a single cell-to-cell movement protein (MP)^1^ to execute all these intracellular and intercellular functions, as typified by the 30-kDa MP encoded by the Tobamovirus *Tobacco mosaic virus* (TMV). TMV, like most RNA viruses, multiplies solely in the cytoplasm of infected cells, and it has been the model for understanding how a single MP functions (Lazarowitz and Beachy, 1999; Nelson and Citovsky, 2005; Verchot-Lubicz et al., 2010). Despite much effort in the past 25 years to delineate the strategies employed by different plant viruses to transport their genomes within and between cells (Harries et al., 2010; Verchot-Lubicz et al., 2010; Harries and Ding, 2011), the key challenge in the field remains to define how movement proteins transport virus genomes to PD and, once there, how they mechanistically alter PD gating. Recent studies in our lab identified the plant synaptotagmin SYTA as a potential key regulator of MP action in modifying PD permeability (Lewis and Lazarowitz, 2010).

Synaptotagmins (SYTs) are a large family of evolutionarily conserved single-pass transmembrane proteins that have been well characterized in animals due to their essential roles in regulating neurotransmitter release and hormone secretion in nerve or neuroendocrine cells (Chapman, 2008; Moghadam and Jackson, 2013). Encoded by a family of at least 17 genes in mammals, and with three synaptotagmins (SYTs 1, 4, and 7) also present in *C. elegans* and Drosophila, all SYTs have a characteristic conserved domain structure: an uncleaved signal peptide that overlaps with a short N-terminal transmembrane domain (TM), followed by a variable domain (VD) and a cytoplasmic C-terminal region that contains tandem C2 Ca^2+^/lipid-binding domains called C2A and C2B (Chapman, 2008; Moghadam and Jackson, 2013). Mammals also encode three related extended synaptotagmins (E-SYTs^2^), which have additional C2 domains and are ubiquitously expressed. While yeast do not encode classical SYTs, they do encode tricalbins, which are E-SYT orthologs that have three C2 domains. Recent studies in mammalian cells and yeast show that E-SYTs act as tethers, along with other proteins, to mediate the formation of contact sites between the endoplasmic reticulum (ER) and plasma membrane (Manford et al., 2012; Toulmay and Prinz, 2012; Giordano et al., 2013).

Mammalian SYTs bind Ca^2+^ and interact with acidic phospholipids and the core SNARE proteins of the membrane fusion machinery to regulate vesicle fusion at the plasma membrane for exocytosis of neurotransmitters or hormones. A variety of studies, mainly on mammalian SYT1, the first identified family member, suggest that synaptotagmins act as Ca^2+^ sensors to regulate fusion pore stability and thereby mediate rapid and synchronous exocytosis (Chapman, 2008; Moghadam and Jackson, 2013). As additional family members were characterized, it became clear that SYTs vary in their affinity for Ca^2+^, binding Ca^2+^ with low (SYTs 1, 2, and 3), intermediate (SYTs 5, 6, 9, and 10) or high affinity (SYT7), or not binding Ca^2+^ at all (>50% of the metazoan SYTs, including SYT4). Although a complete mechanistic understanding is still lacking, the emerging picture is that this functional variation among SYTs, coupled with their likely acting as dimers, may be important in SYTs regulating the choice between transient pore opening to favor the rapid release of small molecules, and full fusion of the vesicle and plasma membranes to release larger molecules (Chapman, 2008; Moghadam and Jackson, 2013). Such functional diversity could be important in fine tuning cellular response to Ca^2+^ signals of varying strength and duration. It is also now apparent that SYTs are found in a variety of cell types, where they can regulate exo/endocytic events beyond synaptic vesicle exocytosis and retrieval, or hormone release, such as Ca^2+^-dependent release of lysosomal proteins or Ca^2+^-triggered exocytosis-mediated membrane repair in non-specialized cell types (Chakrabarti et al., 2005; Becker et al., 2009).

Metazoan SYT1 is the most extensively characterized synaptotagmin. SYT1 is widely expressed in neurons and neuroendocrine cells, where it localizes to synaptic vesicles or large dense core vesicles to tightly couple Ca^2+^ entry with vesicle docking and fusion to trigger rapid exocytosis of neurotransmitters or hormones (so-called synchronous release) (Chapman, 2008; Moghadam and Jackson, 2013). Key to its functions are the tandem C2 domains, which bind Ca^2+^ and interact with effector SNAREs and phospholipids to trigger membrane fusion (Hui et al., 2006; Lee et al., 2010). Studies suggest that the SYT1 C2A and C2B domains, in response to binding Ca^2+^, shallowly insert into the inner leaflet of the plasma membrane to induce local membrane curvature, which favors lipid mixing and leads to membrane fusion (Martens et al., 2007; Hui et al., 2009; McMahon et al., 2010). In addition to exocytosis, SYT1 and SYT4 also regulate endocytosis, in a Ca^2+^-dependent manner, to retrieve membrane from the cell surface and maintain homeostasis (Yao et al., 2012b). Detailed studies of SYT1 activity using missense mutations in C2A and C2B that uncouple exocytosis and endocytosis show that Ca^2+^ binding has different roles in the two processes (Yao et al., 2012b). In exocytosis, Ca^2+^ binding by both C2A and C2B is required for the electrostatic switch to allow the surface loops of both domains to interact with SNAREs and cooperatively penetrate the plasma membrane to trigger efficient synchronous neurotransmitter release (Hui et al., 2006; Striegel et al., 2012). However, either C2A or C2B can function as a Ca^2+^ sensor for endocytosis without apparently penetrating the membrane (Yao et al., 2012a).

We and others recently identified synaptotagmins in plants. The model plant *Arabidopsis thaliana* encodes five synaptotagmins (SYT A, B, C, D, and E), all of which are predicted to have the characteristic domain structure conserved among their animal orthologs (Craxton, 2001, 2004; Schapire et al., 2008; Yamazaki et al., 2008; Lewis and Lazarowitz, 2010). Of these, SYTA is the best studied, and has been shown to have important roles in biotic and abiotic stress (Schapire et al., 2008; Yamazaki et al., 2008; Lewis and Lazarowitz, 2010). Our group identified Arabidopsis SYTA in a yeast interactive screen as a protein that interacts with the Begomovirus CaLCuV cell-to-cell MP^CaLCuV^, and we established its role in regulating endocytosis and MP-mediated cell-to-cell transport using both *in vitro* and *in vivo* approaches (Lewis and Lazarowitz, 2010).

When tested in an *in vitro* pulldown assay, SYTA directly interacts with MP^CaLCuV^, and with MP^SqLCV^ encoded by the Begomovirus SqLCV and the 30-kDa MP^TMV^ encoded by the Tobamovirus TMV (Lewis and Lazarowitz, 2010). To establish the role of SYTA in virus movement, we used an Arabidopsis Col-0 SYTA knockdown line *syta-1*, and a SYTA dominant-negative mutant SYTA^ΔC2B^, in which the C2B domain is deleted (Lewis and Lazarowitz, 2010). Consistent with a role in virus movement, CaLCuV systemic infection is significantly delayed and virus disease symptoms are attenuated in *syta-1* vs. wild type Col-0 plants (Lewis and Lazarowitz, 2010). To show that SYTA regulates MP-mediated virus cell-to-cell transport, we used transient expression assays: MP^CaLCuV^ and MP^TMV^ cell-to-cell trafficking via PD is significantly inhibited when each movement protein, tagged with GFP, is biolistically bombarded into leaves from *syta-1* vs. Col-0 plants, or is co-expressed with SYTA^ΔC2B^ in *Nicotiana benthamiana* leaf epidermal cells using agroinfiltration (Lewis and Lazarowitz, 2010). Using GFP fusions of SYTA and SYTA^ΔC2B^, we also showed that SYTA localizes to plasma membrane-derived endosomes in *N. benthamiana* protoplasts, and we and others found SYTA on endosomes and at discrete areas along the plasma membrane in leaf epidermal cells (Schapire et al., 2008; Lewis and Lazarowitz, 2010). In contrast, SYTA^ΔC2B^ localizes to the plasma membrane in protoplasts, and is not found on endosomes; and it blocks the formation of plasma membrane-derived endosomes and endosomal recycling (Lewis and Lazarowitz, 2010). Our functional studies further demonstrate that SYTA^ΔC2B^ must be at the plasma membrane to interfere with MP^TMV^ or MP^CaLCuV^ cell-to-cell trafficking. Thus, SYTA regulates endocytosis and MP-mediated trafficking of plant virus genomes through PD, and our findings suggest that distinct virus movement proteins transport their cargos to PD for cell-to-cell spread via an endocytic recycling pathway (Lewis and Lazarowitz, 2010).

While all plant viruses exploit PD for virus genome cell-to-cell transport, research on a range of diverse viruses has led to a variety of models for how different plant viruses transport their genomes within and between cells, and how this intracellular and intercellular genome trafficking may be coordinated (Harries et al., 2010; Verchot-Lubicz et al., 2010; Harries and Ding, 2011). Our finding that SYTA regulates MP-mediated transport via PD for viruses as distinct as CaLCuV and TMV suggests that SYTA may have a central role in regulating cell-to-cell movement for a range of diverse plant viruses. To test this hypothesis, we have used infectivity assays and MP-based cell-to-cell trafficking assays in our Arabidopsis Col-0 *syta-1* knockdown line vs. wild type (wt) plants to examine the role of SYTA in the cell-to-cell movement of additional plant viruses that employ different modes of movement, namely the Potyvirus *Turnip mosaic virus* (TuMV), the Caulimovirus *Cauliflower mosaic virus* (CaMV) and the Tobamovirus *Turnip vein clearing virus* (TVCV), which, unlike TMV, efficiently infects Arabidopsis. As reported here, we found that both TuMV and TVCV systemic infection were delayed, and the cell-to-cell trafficking of the their respective movement proteins P3N-PIPO and MP^TVCV^, each tagged with GFP, were inhibited in *syta-1* vs. wild type Col-0 plants. In contrast, CaMV systemic infection was not inhibited in *syta-1*. Our studies show that SYTA is a key regulator of plant virus intercellular movement, being necessary for the ability of diverse cell-to-cell movement proteins encoded by Begomoviruses (MP^CaLCuV^), Tobamoviruses (30K MP^TVCV^ and MP^TMV^) and Potyviruses (P3N-PIPO^TuMV^), but not Caulimoviruses (MP^CaMV^), to alter PD to mediate virus cell-to-cell spread.

## Materials and methods

### Plant material and viruses

*A. thaliana* ecotype Col-0 plants were grown in growth rooms or chambers at 22C under 16 h/8 h light/dark cycle for CaLCuV, TVCV, and TuMV infectivity assays, and 8 h/16 h light/dark cycle for CaMV infectivity and for movement protein trafficking assays (Lewis and Lazarowitz, 2010). For transient expression studies using agroinfiltration, *N. benthamiana* plants were grown and maintained under greenhouse conditions of 25C with 16 h supplemental lighting (Levy et al., 2013). Genomic clones of CaLCuV A and B components, TuMV (pGreen-TuMV), TVCV (p50TVCV) and CaMV (pCa122) have all been described, as has the Col-0 T-DNA insertion line *syta-1* (Kobayashi et al., 2002; Carvalho et al., 2004; Dunoyer et al., 2004; Lewis and Lazarowitz, 2010; Levy et al., 2013).

### Infectivity assays

Agroinoculation of Arabidopsis Col-0 plants with CaLCuV A and B genomic components, each cloned in the binary vector pCB301, has been described (Carvalho et al., 2004). We used the same protocol for TuMV infectivity assays. In brief, cultures of *Agrobacterium tumefaciens* GV3101 containing the appropriate infectious clones were grown in LB media at 28C for 16 h, diluted 1:20 into fresh LB and incubated an additional ~20 h. The first two true leaves of *A. thaliana* 12-da-old seedlings were cut transversally with a razor blade and dipped for 5 min into the *A. tumefaciens* GV3101 cultures harboring CaLCuV (A and B clones mixed) or pGreen-TuMV, each of which had been concentrated to OD_600_ of 0.5. Plants were maintained at 22C under 16 h/8 h light/dark cycle and scored for viral disease symptoms. For TVCV infectivity assays, Col-0 plants at the 8-leaf stage were rub inoculated with 10 μl of a standardized amount of leaf extract from *N. benthamiana* plants that had been infected with RNA transcripts of p50TVCV, as previously described (Levy et al., 2013). Infectivity was determined based on the levels of TVCV CP in extracts of systemic leaves (0.5 g tissue/ml buffer), as analyzed by SDS-PAGE and Coomassie blue staining (Levy et al., 2013). CaMV infectivity assays were done by bombarding leaves of 3-wk-old *Arabidopsis* plants with pCa122 as previously described (Kobayashi et al., 2002). To quantify CaMV DNA levels in systemic leaves, primers 5′-ATGGCCGAATCAATTTTAGACAGGACC-3′ and 5′-CGATGTTGAGCATTCCCATAGACGTTT-3′ were used for semi-quantitative PCR (sqPCR) analysis to amplify the first 455 nucleotides of the CaMV coat protein (*CP*) gene. For an internal loading control, we used primers 5′-CATGGAGTTGTCAGTGAAATGGGC-3′ and 5′-GTCTTTGGCCATAGGTACATG-3′ to sqPCR amplify nucleotides 992–1554 of the *SYTB* gene sequence. DNA was extracted from mock-inoculated control plants or from CaMV systemically infected leaves at 21 da post inoculation, as previously described (Lewis and Lazarowitz, 2010).

### Movement protein trafficking assays

Cell-to-cell movement of MP^CaLCuV^, MP^TVCV^ and TuMV P3N-PIPO, and tubule formation by MP^CaMV^, was assayed by biolistic bombardment of Arabidopsis leaves with plasmids that expressed GFP-fusions of each movement protein under the control of the CaMV 35S promoter. The plasmids pTEX::MP^CaLCuV^-GFP, and p35S::P3N-PIPO-GFP cloned in the plant expression vector pJ4GFP-XB, have been described (Lewis and Lazarowitz, 2010; Vijayapalani et al., 2012). We used Gateway technology to clone MP^CaMV^ and MP^TVCV^ C-terminal fusions to GFP (MP^CaMV^-GFP and MP^TVCV^-GFP). The coding sequence for MP^CaMV^ was PCR amplified using primers 5′-GGGGACAAGTTTGTACAAAAAAGCAGGCTTTATGGATTTGTATCCAGAAG-3′ and 5′-GGGGACCACTTTGTACAAGAAAGCTGGGTCTTCTCCACAGATTTCTTTTAA-3. Primers 5′-GGGGACAAGTTTGTACAAAAAAGCAGGCTTTATGTCGATAGTCTCGTACGAACC-3′ and 5′-GGGGACCACTTTGTACAAGAAAGCTGGGTCAGCATTGGTATGGGCTCTGC-3′ were used to PCR amplify the MP^TVCV^ coding sequence. PCR products were cloned into the entry vector pDONR207 and then recombined into the destination vector pSITE2NB (Chakrabarty et al., 2007) to create pSITE2::MP^CaMV^-GFP and pSITE2::MP^TVCV^-GFP. To assess movement protein cell-to-cell trafficking or tubule formation, detached leaves from 4-wk-old Arabidopsis wild type Col-0 or *syta-1* plants were biolistically bombarded with pTEX::MP^CaLCuV^-GFP, TuMV p35S::P3N-PIPO-GFP, pSITE2::MP^CaMV^-GFP or pSITE2::MP^TVCV^-GFP using the Helios Gene Gun (Bio-Rad, Hercules, CA), as described (Ueki et al., 2009). We used confocal imaging to count or examine cells in individual fluorescent foci at different time points between 8 and 48 h post bombardment.

### Statistical analyses

These were done using JMP Pro 0.0.2 and SAS software (SAS, Cary, NC). Local cell-to-cell spread was analyzed using X^2^ analysis, and the Wilcoxon test was applied to infectivity assays.

### Microscopy

Confocal laser scanning microscopy (CLSM) was performed using a Leica TCS-SP5 microscope (Leica Microsystems, Exton, PA) with a 63x/NA1.2 water immersion objective. GFP fluorescence was excited with a 488-nm argon laser, and emission was detected at 500–530 nm.

## Results

### TuMV and TVCV infection is delayed in a SYTA knockdown line

We previously reported that Begomovirus CaLCuV systemic infection is significantly delayed in the Arabidopsis Col-0 T-DNA insertion mutant line *syta-1* (Sail 775A08) (Lewis and Lazarowitz, 2010). The T-DNA in this line is inserted just downstream of the SYTA C_2_B coding region. This insertion produces a functional chimeric SYTA protein (the signal peptide, and TM, VD, and C2A and C2B domains all remain intact) that is slightly smaller (59.1 kDa) than wild type SYTA (61.7 kDa), and this truncated form of SYTA protein in *syta-1* accumulates to only ~10% the level of full-length SYTA produced in wild type Col-0 (Lewis and Lazarowitz, 2010). Despite this, our homozygous *syta-1* lines do not exhibit overt phenotypes, which makes this SYTA knockdown mutant ideally suited for virus infectivity and movement protein trafficking studies (Lewis and Lazarowitz, 2010). CaLCuV systemic spread is delayed from 1 to 3 days, and viral disease symptoms are attenuated, in *syta-1* vs. wild type Col-0 plants, based on the onset of appearance of viral disease symptoms and PCR analysis to assess viral genome accumulation in systemic leaf extracts. CaLCuV infectivity levels are also inhibited ~10 to ~30% in *syta-1* (Lewis and Lazarowitz, 2010).

To assess whether SYTA is more generally required for infection and spread of diverse plant viruses, we inoculated wild type Col-0 and two separate homozygous *syta-1* segregant lines [lines 1 and 16, see Lewis and Lazarowitz (2010)] with the Potyvirus TuMV or the Tobamovirus TVCV, and analyzed virus infectivity levels and the spread of systemic infection. CaLCuV is a single-stranded DNA (ssDNA) virus that replicates in the nucleus. Both TuMV and TVCV are positive-sense RNA (+RNA) viruses that replicate in the cytoplasm (Lazarowitz, 2001), and our recent BiFC studies show that MP^TVCV^ and SYTA directly interact *in planta* (A. Levy, J. Zheng and SGL, submitted). TuMV infectivity and systemic spread were monitored by the development of viral disease symptoms. Because TVCV produces mild disease symptoms (variable degrees of stunted and bent stems) on Arabidopsis Col-0, we assessed TVCV infectivity and spread by SDS-PAGE analysis to determine the accumulation of viral coat protein (CP) in systemic leaves at different days following inoculation (Levy et al., 2013).

**Figure 1:**
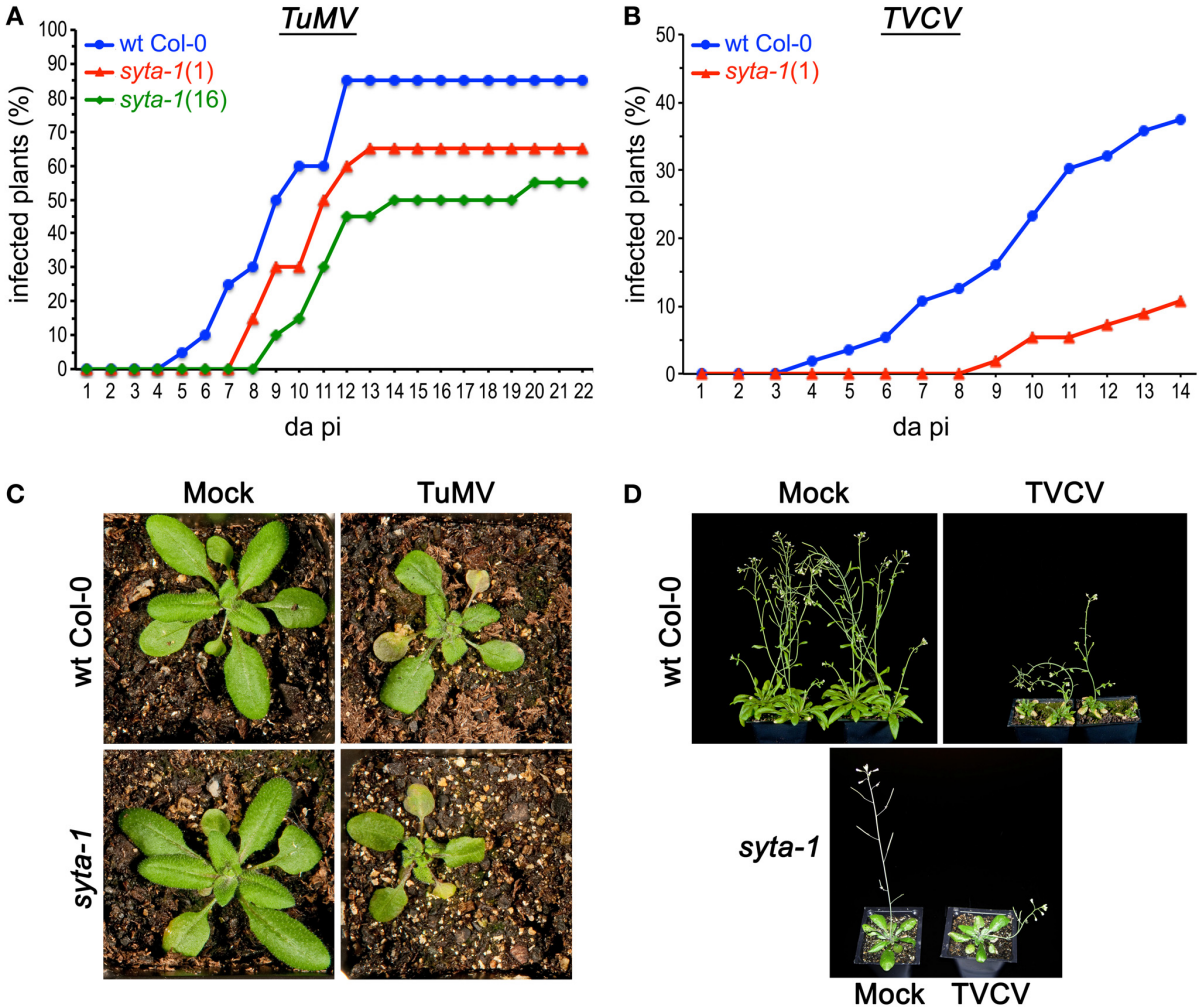
**TuMV and TVCV infections are delayed in *syta-1*. (A)** Time course of appearance of disease symptoms on wild type (wt) Arabidopsis Col-0 plants (blue), and *syta-1* mutant lines 1 (red) and 16 (green) inoculated with equal amounts of TuMV (Table 1, Trial 2). **(B)** Development of systemic disease as assessed by viral CP accumulation in Col-0 plants (blue) and *syta-1* mutant line 1 (red) inoculated with equal amounts of TVCV (Table 2, Trial 3). **(C)** TuMV or mock inoculated wild type Col-0 or *syta-1* plants at 16 da post inoculation (pi). **(D)** TVCV or mock inoculated wild type Col-0 plants at 30 da post inoculation or *syta-1* plants at 14 da post inoculation (pi).

**Figure 2:**
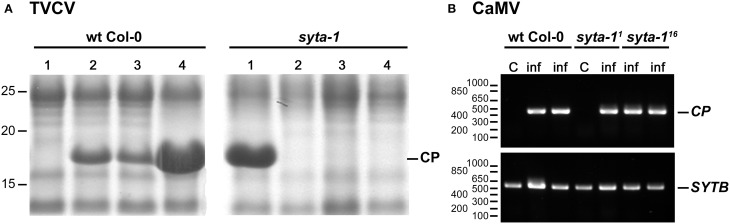
**TVCV, but not CaMV, systemic spread is delayed in *syta-1*. (A)** Coomassie blue-stained SDS-PAGE gels of systemic leaf extracts from four TVCV-inoculated Arabidopsis wt Col-0 or *syta-1* (line 1) plants at 10 da post-inoculation. Positions of TVCV CP (17.5 kDa) and molecular weight markers (kDa) are indicated. **(B)** Semi-quantitative PCR analyses of CaMV genome DNA in systemic leaf extracts from mock inoculated control (C) or CaMV infected (inf) plants at 21 da post-inoculation. Shown are agarose gel analyses of extracts from Arabidopsis wt Col-0 plants and the two independent mutant lines *syta-1* (line 1) (*syta-1^1^*) and *syta-1* (line 16) (*syta-1^16^*). Positions of PCR products of CaMV *CP* gene and *SYTB* gene (internal loading control), and of molecular weight markers (nt) are indicated. Equal amounts of systemic leaf extracts were analyzed in each gel in panels **(A)** and **(B)**.

As we found for CaLCuV, infection by both TuMV and TVCV progressed more slowly in *syta*-1 when compared to wild type Col-0 plants: the onset of systemic infection was delayed, and infectivity levels were lower in *syta-1* lines (Figure 1A; Tables 1, 2). TuMV systemic disease symptoms in wild type Col-0 were first evident at 5–6 da post inoculation, and 75–100% of inoculated wild type plants became infected. In contrast, the onset of TuMV disease symptoms was delayed from 1 to 3 da in our *syta-1* knockdown lines, and infectivity was decreased to ~60–85% of the levels in wild type Col-0 (50–85% of the inoculated plants became infected), although there was no apparent attenuation of viral disease symptoms (Figures 1A,C; Table 1). TVCV systemic infection, based on the accumulation of viral CP, was also first evident at 5–6 da post inoculation in wild type Col-0, with ~30–45% of inoculated wild type plants being systemically infected by 16 da post-inoculation. In contrast, the onset of TVCV systemic infection was delayed from 1 to 4 da in *syta-1* plants and infectivity was decreased to only ~30–50% of the levels in wild type Col-0, based again on the accumulated levels of TVCV CP in systemic leaf extracts (11–20% of inoculated plants became systemically infected) (Figures 1B,D, 2A; Table 2). Furthermore, the levels of TVCV CP in systemic leaf extracts were, on average, lower in *syta-1* vs. wt Col-0 plants on the same days post-inoculation (Figure 2A, and data not shown). As with TuMV, TVCV disease symptoms appeared to be the same in wild type Col-0 and *syta-1* plants at all stages of infection (Figure 1D and data not shown). Thus, as we had found for CaLCuV, SYTA was necessary for TuMV and TVCV systemic spread and infection in Arabidopsis, consistent with SYTA being important for the cell-to-cell movement of these two +RNA viruses.

**Table 1:** **TuMV Infectivity on *syta-1* and wild type *Arabidopsis* Col-0^a^**.

**da post infection (pi)**																						
		**4**	**5**	**6**	**7**	**8**	**9**	**10**	**11**	**12**	**13**	**14**	**15**	**16**	**17**	**18**	**19**	**20**	**21**	**23**	**24**	**25**
1	*syta-1*	0	0	5	15	30	40	55	60	70	75	80	80	85	85	85	85	85				
	Col-0	0	0	21	53	74	95	100	100	100	100	100	100	100	100	100	100	100				
	Mock	0	0	0	0	0	0	0	0	0	0	0	0	0	0	0	0	0				
2	*syta-1* (1)	0	0	0	0	15	30	30	50	60	65	65	65	65	65	65	65	65	65			
	*syta-1* (16)	0	0	0	0	0	10	15	30	45	45	50	50	50	50	50	50	55	55			
	Col-0	0	5	10	25	30	50	60	60	85	85	85	85	85	85	85	85	85	85			
	Mock	0	0	0	0	0	0	0	0	0	0	0	0	0	0	0	0	0	0			
3	*syta-1*	0	0	0	2	11	30	45	47	49	57	57	60	66	70	72	72	74	74	74	74	74
	Col-0	0	0	8	19	46	56	67	71	77	81	81	81	81	81	83	83	83	88	88	88	88
	Mock	0	0	0	0	0	0	0	0	0	0	0	0	0	0	0	0	0	0	0	0	0
4	*syta-1*	0	0		0	0	0	5		30	30	41	43	43	48	48	52	52	52	52	57	57
	Col-0	0	0		2	2	2	2		25	32	50	57	61	68	70	70	73	73	73	73	73
	Mock	0	0		0	0	0	0		0	0	0	0	0	0	0	0	0	0	0	0	0
5	*syta-1*	0	0		0		13	16	22	28	38	38	38	44		47	47		50	50	50	53
	Col-0	0	0		18		52	61	67	67	67	70	73	73		73	76		79	82	85	85
	Mock	0	0		0		0	0	0	0	0	0	0	0		0	0		0	0	0	0

a *Symptomatic plants as percentage of total inoculated plants (40–60 plants of each genotype per assay). Nonparametric survival analysis with right censoring (Wilcoxon test statistic) is p < 0.001 for the five trials shown. Unless noted otherwise, syta-1 (line 1) was used for infectivity assays*.

**Table 2:** **TVCV Infectivity on *syta-1* and wild type *Arabidopsis* Col-0^a^**.

**da post infection (pi)**															
		**3**	**4**	**5**	**6**	**7**	**8**	**9**	**10**	**11**	**12**	**13**	**14**	**15**	**16**
1	*syta-1*	0	0	0	0	2	2	2	4	4	7	7	9	11	11
	Col-0	0	0	0	4	4	7	7	11	14	18	21	25	27	29
	Mock	0	0	0	0	0	0	0	0	0	0	0	0	0	0
2	*syta-1*	0	0	0	0	0	2	4	4	5	5	11	11	14	20
	Col-0	0	0	2	4	4	8	12	18	24	26	32	36	38	42
	Mock	0	0	0	0	0	0	0	0	0	0	0	0	0	0
3	*syta-1*	0	0	0	0	0	0	0	0	2	5	5	7	9	11
	Col-0	0	0	0	2	4	5	11	13	16	23	30	32	36	38
	Mock	0	0	0	0	0	0	0	0	0	0	0	0	0	0

a *Symptomatic plants as percentage of total inoculated plants (50–56 plants of each genotype per assay). Nonparametric survival analysis with right censoring (Wilcoxon test statistic) is p < 0.05 for the three trials shown*.

### SYTA regulates the cell-to-cell trafficking of the TuMV and TVCV movement proteins

The Begomovirus MP^CaLCuV^ and MP^SqLCV^, and the Tobamovirus MP^TMV^, directly bind to SYTA *in vitro*, which suggests a potential role for SYTA in virus movement, and we have established that SYTA regulates the cell-to-cell trafficking of MP^CaLCuV^ and MP^TMV^, when each is transiently expressed as functional fluorescently-tagged proteins in *N. benthamiana* or Arabidopsis leaf epidermal cells (Lewis and Lazarowitz, 2010). Given our previous findings, the delay we observed in the onset of TVCV disease symptoms and the inhibition of infectivity in *syta-1* plants were not unexpected. To directly determine whether SYTA regulated TVCV and TuMV cell-to-cell movement, we used our biolistic bombardment assay to transiently express functional GFP fusions of MP^TVCV^ and TuMV P3N-PIPO (hereafter P3N-PIPO^TuMV^) in Arabidopsis *syta-1* compared to wild type Col-0 plants (Lewis and Lazarowitz, 2010). We used the Golgi marker t-TM-GFP, which contains the transmembrane domain from rat sialotransferase and does not traffic between cells, to determine an optimal amount of plasmid DNA that would produce foci in which ≥95% contained a single fluorescent cell and ≤5% contained only 2 fluorescent cells at 16–20 h post bombardment (Table 3) (Lewis and Lazarowitz, 2010). We also determined the earliest time (~8–20 h post bombardment, depending on the MP) at which each MP would be expressed in isolated single cells (≥95% of foci, with ≤5% having two adjacent GFP-labeled cells) and then assessed MP cell-to-cell trafficking over time using CLSM. We included GFP-MP^CaLCuV^ as a positive control (Lewis and Lazarowitz, 2010). As we previously reported (Lewis and Lazarowitz, 2010; Levy et al., 2013), we did not observe any qualitative differences at the imaging resolution of these MP trafficking assays in wt Col-0 vs. *syta-1* plants for each MP tested. Our higher resolution studies of MP^TVCV^ also show quantitative, but no qualitative differences, for MP^TVCV^-GFP expressed in wt Col-0 vs. *syta-1* plants (A. Levy, J. Zheng and SGL, submitted).

**Table 3:** **MP cell-to-cell trafficking in *syta-1* and wild type Col-0**.

**MP**	**Trial**	**Line**	**Total foci (cells)**	**1 cells (%)^a^**	**≥2cells (%)^a^**	***P*(χ^2^)^b^**
GFP-MP^CaLCuV^c^^	1	wt	45 (165)	28.9	71.1	<0.0001
		*syta-1*	46 (136)	50.0	50.0	
	2	wt	33 (158)	21.2	78.8	
		*syta-1*	32 (94)	46.9	53.1	
	3	wt	33 (150)	12.1	87.8	
		*syta-1*	30 (74)	56.7	43.3	
MP^TVCV^-GFP^c^	1	wt	25 (124)	8.0	92.0	<0.0001
		*syta-1*	30 (97)	30.0	70.0	
	2	wt	15 (92)	6.7	93.3	
		*syta-1*	25 (68)	58.8	41.2	
	3	wt	17 (77)	13.8	86.2	
		*syta-1*	20 (64)	30.0	70.0	
	4	wt	29 (117)	13.8	86.2	
		*syta-1*	26 (69)	34.6	65.4	
P3N-PIPO^TuMV^-GFP^c^	1	wt	21 (44)	38.1	61.9	<0.0001
		*syta-1*	17 (23)	64.7	35.3	
	2	wt	26 (48)	42.3	57.7	
		*syta-1*	28 (35)	75.0	25.0	
	3	wt	27 (58)	40.7	59.3	
		*syta-1*	29 (33)	89.7	10.3	
	4	wt	27 (47)	48.1	51.9	
		*syta-1*	26 (30)	88.5	11.5	
t-TM-GFP^d^	1	wt	57 (60)	94.7	5.3^d^	----
	2	wt	58 (60)	96.6	3.4^d^	
	3	wt	40 (41)	97.5	2.5^d^	
	4	wt	40 (40)	100.0	0.0^d^	

a *Percent fluorescent cells where each MP was in a single cell (1 cell), or moved to 2 or more cells (≥2)*.

b *χ^2^ test for cell-to-cell movement of each MP bombarded into syta-1 vs. wild type Col-0 leaves for all three or four trails shown*.

c *Each MP was assayed for cell-to-cell movement at the following times post bombardment: MP^CaLCuV^ 36–46 h, MP^TVCV^ 24 h, P3N-PIPO^TuMV^ 48 h, MP^CaMV^ 24 h*.

d *Golgi marker: transmembrane domain (TM) of rat sialotranferase fused to GFP. No difference was found in the percentage of foci with 1 vs. 2 labeled cells for syta-1 vs. wild type Col-0. Foci with >2 adjacent t-TM-GFP-labeled cells were never observed. Foci were imaged at 12–24 h post bombardment*.

**Figure 3:**
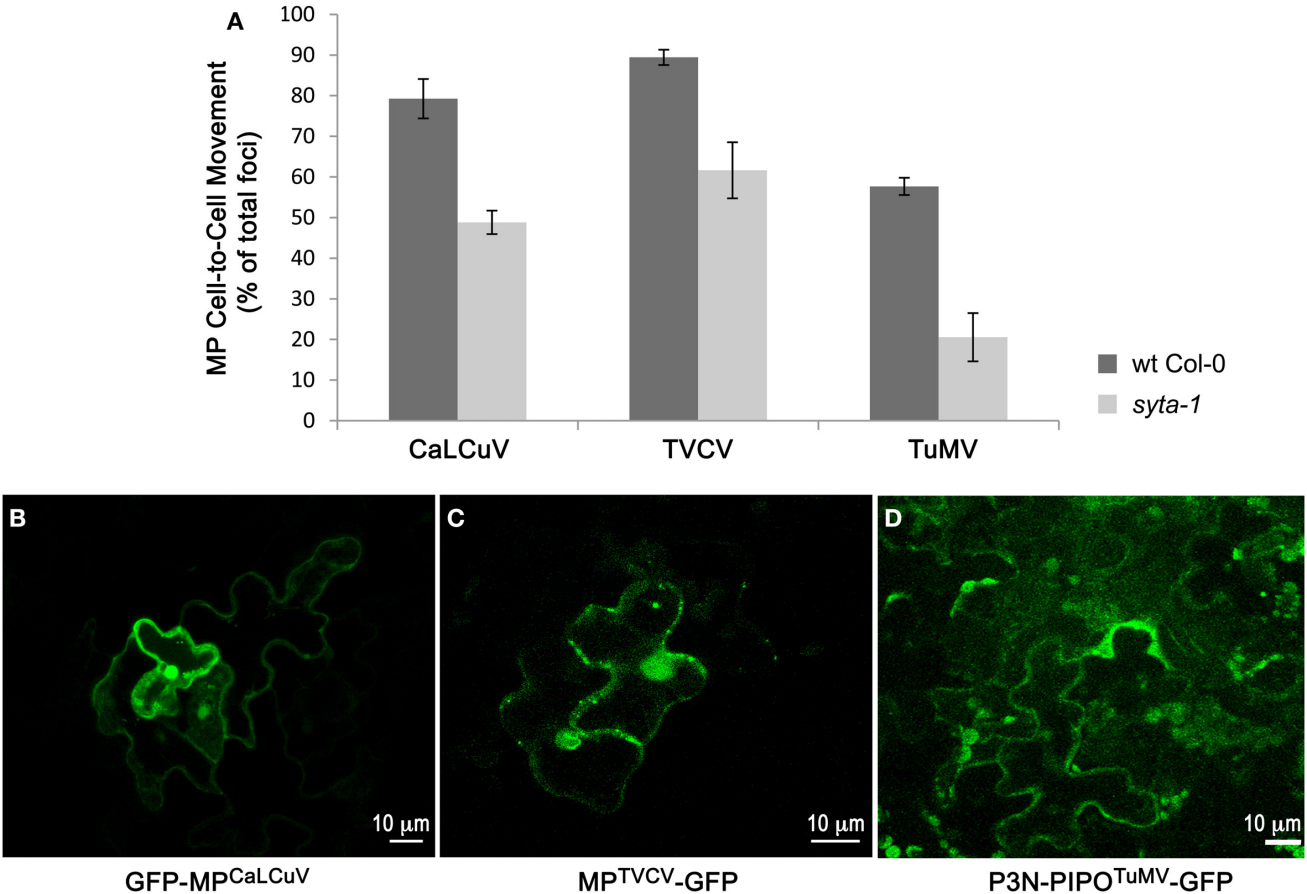
**Cell-to-cell trafficking of MP^CaLCuV^, MP^TVCV^ and P3N-PIPO^TuMV^ is inhibited in *syta-1*. (A)** Percent of total foci in which MP^CaLCuV^, MP^TVCV^ or P3N-PIPO^TuMV^ moved to 2 or more cells in wild type (wt) Col-0 or *syta-1* (line 1) plants. Shown are averages from 3 (MP^CaLCuV^) or 4 (MP^TVCV^ and P3N-PIPO^TuMV^) independent trials (Table 3). **(B–D)** Images of typical individual foci on wt Col-0 bombarded with GFP-MP^CaLCuV^, MP^TVCV^-GFP and P3N-PIPO^TuMV^-GFP show cell-to-cell trafficking at 40 h (MP^CaLCuV^), 24 h (MP^TVCV^) or 48 h (P3N-PIPO^TuMV^) post bombardment.

The cell-to-cell trafficking of MP^TVCV^-GFP and P3N-PIPO^TuMV^-GFP were both significantly inhibited in *syta-1* knockdown lines as compared to wild type Arabidopsis Col-0 (Figure 3, Table 3). Similar to MP^TMV^-GFP, MP^TVCV^-GFP moved into surrounding cells in 86–93% of the foci in wild type Col-0 plants. In contrast, MP^TVCV^-GFP spread to surrounding cells only ~40–70% of the time in *syta-1* (Figures 3A,C, Table 3). P3N-PIPO^TuMV^-GFP moved into surrounding cells in ~50–60% of the foci in wild type plants, similar to what was previously reported in *N. benthamiana* (Vijayapalani et al., 2012). In contrast, P3N-PIPO^TuMV^-GFP spread to surrounding cells only 10–35% of the time in *syta-1* (Figures 3A,D, Table 3). Similar to what we previously reported, GFP-MP^CaLCuV^ moved into surrounding cells in ~70–88% of foci in wild type Col-0 compared to only ~43–50% of foci in *syta-1* plants (Figures 3A,B, Table 3). Consistent with our infectivity studies, these results show that SYTA regulates the cell-to-cell trafficking of MP^TVCV^ and P3N-PIPO^TuMV^ via PD, as well as of MP^CaLCuV^ and MP^TMV^ (Lewis and Lazarowitz, 2010).

### SYTA is not required for CaMV infectivity

We further examined whether SYTA might have a role in the infectivity and movement of the Caulimovirus CaMV, which is a pararetrovirus. The CaMV dsDNA genome is transcribed into a 35S polycistronic mRNA in the nucleus by host cell RNA polymerase II. Encapsidation in the cytoplasm of this 35S +RNA and the viral-encoded reverse transcriptase activates the reverse transcriptase to synthesize the CaMV dsDNA genome within assembled virus particles. Thus, unlike the Begomoviruses, Tobamoviruses, and Potyviruses, CaMV moves cell-to-cell as virus particles, aided by a Virion-Associated Protein (VAP) that associates with viral CP to translocate virions across PD through tubules formed by MP^CaMV^ (Lazarowitz, 2001; Stavolone et al., 2005).

**Figure 4:**
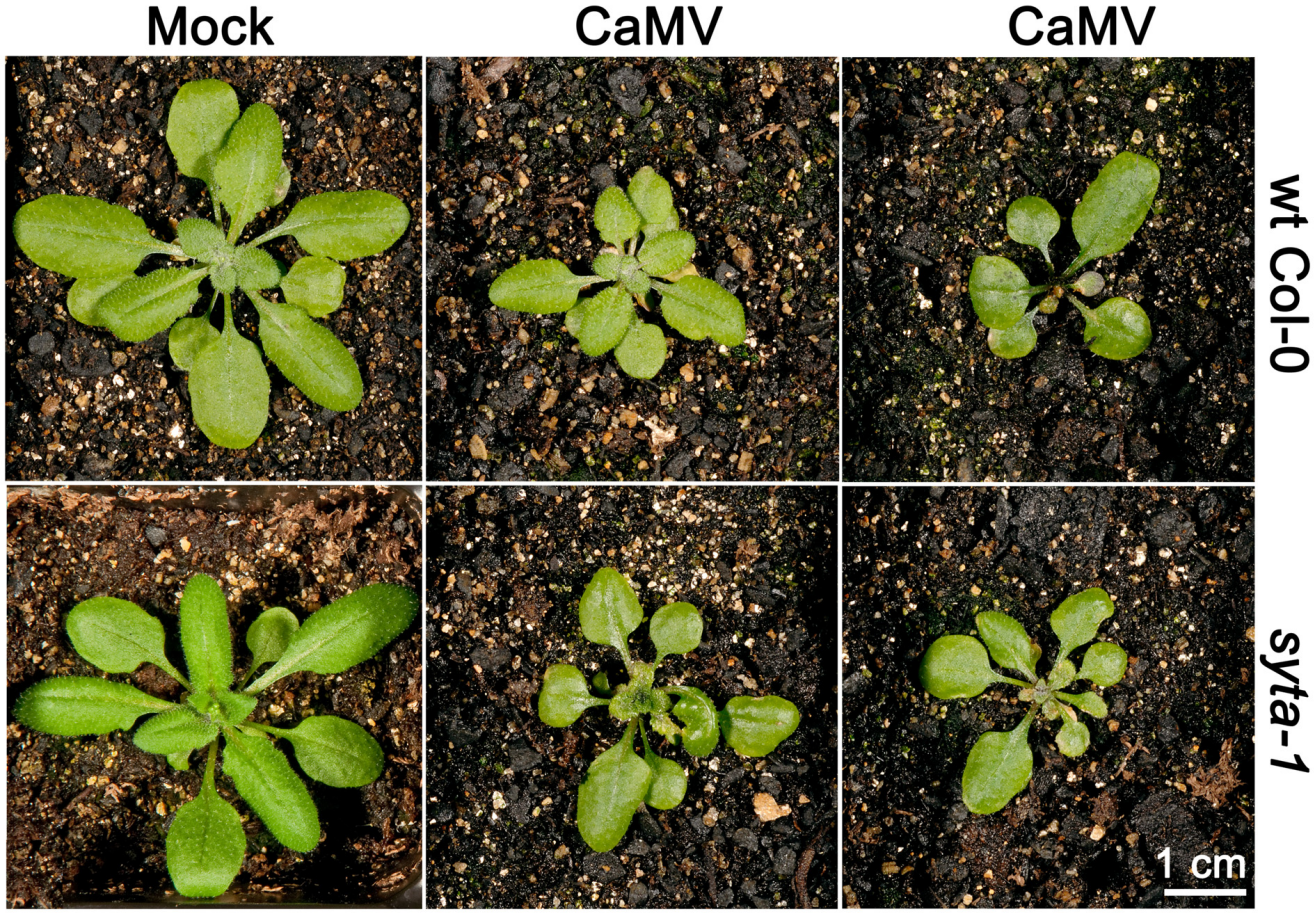
**CaMV produces typical disease symptoms in *syta-1* plants**. CaMV or mock inoculated wild type Col-0 or *syta-1* plants at 16 da post inoculation. Two typical examples of CaMV-infected plants are shown for wt Col-0 and *syta-1* plants.

We tested CaMV infectivity on our *syta-1* knockdown lines compared to wild type Col-0 plants. Based on the appearance of typical viral systemic disease symptoms and quantifying CaMV DNA levels in systemically infected leaves, CaMV infectivity levels and systemic spread on *syta-1* compared to wild type Col-0 plants were not statistically different (Figures 2B, 4, Table 4). In contrast to TuMV and TVCV (Figure 1, Tables 1, 2), and to CaLCuV (Lewis and Lazarowitz, 2010), CaMV systemic disease symptoms first appeared at the same time on both *syta-1* and wild type plants, and CaMV infection progressed at the same rate, attaining levels up to 100% infectivity in both *syta-1* and wt Col-0 plants. Fitting with this, CaMV DNA accumulated to the same levels in systemically infected leaves from both *syta-1* and wt Col-0 plants.

**Table 4:** **CaMV Infectivity on *syta-1* and wild type *Arabidopsis* Col-0^a^**.

**da post infection (pi)**																					
		**6**	**7**	**8**	**9**	**10**	**11**	**12**	**13**	**14**	**15**	**16**	**17**	**18**	**19**	**20**	**21**	**22**	**23**	**24**	**25**
1	*syta-1*	0	0	0	0	0	0	0	14	47	49	53	67	77	77	79	84	86	86	91	91
	Col-0	0	0	0	0	0	0	2	9	43	57	77	91	95	95	95	98	98	98	100	100
	Mock	0	0	0	0	0	0	0	0	0	0	0	0	0	0	0	0	0	0	0	0
2	*syta-1*	0	5	24	38	74	88	88	98	100											
	Col-0	0	20	45	55	90	95	95	100	100											
	Mock	0	0	0	0	0	0	0	0	0											
3	*syta-1*	0	0	0	5	5	13	21	32	37	50	53	55	61	61	61	66	66			
	Col-0	0	0	0	3	3	16	21	29	39	47	66	68	74	79	79	79	82			
	Mock	0	0	0	0	0	0	0	0	0	0	0	0	0	0	0	0	0			

a *Symptomatic plants as percentage of total inoculated plants (40–44 plants of each genotype per assay). Nonparametric survival analysis with right censoring (Wilcoxon test statistic) is p = 0.87 for the three trials shown*.

MP^CaMV^ forms tubules and does not traffic cell to cell itself. While MP^CaMV^ with an N- or C-terminal GFP tag can form tubules in plant protoplasts, the GFP tag interferes with the formation of the MP^CaMV^-containing tubules required for CaMV cell-to-cell movement when GFP-MP^CaMV^ or MP^CaMV^-GFP is expressed in insect cells or *in planta*, probably due to steric hindrance (Thomas and Maule, 2000). This *in vivo* tubule formation can be restored by mixing GFP-tagged and untagged native MP^CaMV^; but, it is difficult to quantify tubule formation at PD due to the background of GFP-tagged MP^CaMV^ aggregates in the cytoplasm and the low efficiency of tubule formation (Thomas and Maule, 2000; Amari et al., 2010). We attempted to assess whether SYTA affected MP^CaMV^ tubule formation by co-bombarding Arabidopsis leaves with GFP-MP^CaMV^ or MP^CaMV^-GFP mixed with native MP^CaMV^ at ratios of 1:2, 1:1, and 2:1. We observed what appeared to be a small number of MP^CaMV^-labeled tubules formed at the cell periphery in each of the co-bombarded samples, which were similar in appearance to GFP-MP^GFLV^ tubules in leaf epidermal cells (Thomas and Maule, 2000; Amari et al., 2010). We also observed cytoplasmic MP^CaMV^ that likely was the previously reported GFP-tagged MP^CaMV^ aggregates (data not shown) (Thomas and Maule, 2000; Amari et al., 2010). Consistent with our CaMV infectivity assays, we found no obvious differences when we co-bombarded a 1:1 mix of MP^CaMV^-GFP/MP^CaMV^ into leaves from *syta-1* and wild type Col-0 plants. Take together, our findings suggest that, unlike Begomoviruses, Tobamoviruses and Potyviruses, SYTA does not regulate CaMV cell-to-cell movement.

## Discussion

Viral movement proteins are essential to coordinate replication of viral genomes with their directed transport to PD, and to facilitate their transport through PD for cell-to-cell spread. A single MP alone may execute these intracellular and intercellular genome transport functions, as is the case for the Tobamoviruses TMV and TVCV; or the cell-to-cell MP may cooperate with additional viral-encoded proteins to coordinate these events, such as occurs for the Begomovirus CaLCuV, the Potyvirus TuMV, and the Caulimovirus CaMV. Whichever the case, the ultimate path through the cell wall is a common one: all plant viruses exploit PD. Yet, how viral genomes reach PD and, once there, how MPs mechanistically alter PD gating, remain unknown. Our previous studies identified Arabidopsis SYTA as a plant synaptotagmin that interacts with the MPs encoded by the Begomovirus CaLCuV and the Tobamovirus TMV, and showed that SYTA regulates endocytosis and marks an endosomal recycling pathway that we proposed traffics these MPs to PD (Lewis and Lazarowitz, 2010). We have now expanded our analyses to include viruses from other distinct families, namely the Potyvirus TuMV and the Caulimovirus CaMV, as well as a Tobamovirus that infects Arabidopsis (TVCV), in order to test the hypothesis that SYTA has a central role in regulating cell-to-cell movement for a range of diverse plant viruses. Our findings show that SYTA regulates the cell-to-cell movement of TVCV and TuMV, in addition to CaLCuV, despite their distinct modes of movement; but, it does not regulate CaMV movement.

What is the precise role of SYTA in regulating virus movement? Although the mechanistic details remain to be elucidated, the known functions of synaptotagmins, and current knowledge about the different strategies for cell-to-cell movement employed by the plant viruses used in our studies, do provide some clues. While all plant viruses move cell to cell via PD, a variety of schemes have been described for how plant viruses from different families accomplish this feat. These models reflect different viral replication strategies, and whether the virus moves in an encapsidated form (i.e., is CP required for movement) or its MP interacts with additional viral proteins to traffic progeny viral genomes from replication sites to, and through, PD. Based on biophysical considerations, such MP-genome complexes, regardless of whether they contain additional viral-encoded proteins, are too large to freely diffuse from sites of viral replication, be these nuclear or cytoplasmic, through the cytosol to reach PD. Hence, trafficking from replication sites to PD must be an active process that involves the endomembrane network and vesicle transport, and/or the cytoskeletal system (Oparka, 2004; Schoelz et al., 2011).

### Tobamoviruses

The Tobamoviruses TMV and TVCV, with their +RNA genomes that are replicated in the cytoplasm, represent potentially the simplest scenario. These viruses encode a single 30-kDa MP and do not require CP for local cell-to-cell spread. In principle, moving simply the +RNA genome into new cells would suffice to initiate infection. Consistent with this, MP^TMV^ and MP^TVCV^ can bind RNA and localize to PD, and MP^TMV^ was the first movement protein shown to alter PD gating (Wolf et al., 1989; Citovsky et al., 1990; Heinlein et al., 1998; Levy et al., 2013). These findings, together with detailed studies of the subcellular distribution of MP^TMV^ and MP^TVCV^ during the time course of infection, are consistent with the model that the Tobamovirus 30-kDa MP binds progeny +RNA genomes at their site(s) of replication and traffics them to PD, where MP then acts to increase PD permeability and thereby facilitate the transport of viral genomes into neighboring cells to initiate new infections.

A range of studies underscore the importance of the ER and endomembrane system in this process. TMV and TVCV replicate in the cytoplasm at specific replication sites, which are inclusions of viral RNA, MP and replicase that form at cortical ER membrane sites (Heinlein et al., 1998; Schoelz et al., 2011; Levy et al., 2013). Forming viral replication sites in association with host membranes is a common feature of +RNA viruses that infect animals and plants, and is probably a strategy to protect viral genomes from recognition and destruction by host innate defenses (den Boon and Ahlquist, 2010). Both MP^TMV^ and MP^TVCV^ have been shown to associate with ER membranes (Heinlein et al., 1998; Fujiki et al., 2006; Levy et al., 2013), and a variety of studies indicate that this association is necessary for these Tobamovirus MPs to target viral genomes to PD for cell-to-cell spread. Expression of a dominant-negative form of the GTPase Sar1 or low concentrations of Brefeldin A (BFA), both of which disrupt vesicle transport from the ER to the Golgi, do not inhibit MP^TMV^ trafficking to PD or virus infection; but, high concentrations of BFA that disrupt the ER network do prevent MP^TMV^ from reaching PD (Heinlein et al., 1998; Tagami and Watanabe, 2007; Wright et al., 2007). These findings, and mutational analyses of MP^TMV^, lead to the conclusion that although MP^TMV^ does not traffic through the secretory pathway to reach PD, the ER and endomembrane system are necessary for MP^TMV^-genome complexes to target to PD and for TMV cell-to-cell spread (Schoelz et al., 2011). Our demonstration that SYTA and endosomal recycling to the plasma membrane regulate the ability of MP^TMV^ and MP^TVCV^ to reach and alter PD, and TVCV cell-to-cell movement (Lewis and Lazarowitz, 2010) (and our findings here), together with the subcellular distribution of MP^TVCV^ during the course of virus infection and interaction of MP^TVCV^ and SYTA *in planta* (Levy et al., 2013; A. Levy, J. Zheng, and SGL, submitted), lead to the same conclusion for MP^TVCV^. This unifies the findings for TMV and TVCV by showing that the ER and vesicle trafficking are required for both MP^TMV^ and MP^TVCV^ to traffic to PD and for TVCV, as well as TMV, intercellular movement. In addition, functional studies using our dominant negative mutant SYTA^ΔC2B^ show that the SYTA-regulated vesicle trafficking pathway is distinct from the secretory pathway (Lewis and Lazarowitz, 2010). Thus, neither MP^TVCV^ nor MP^TMV^ traffic to PD via the secretory pathway.

In contrast to the common requirement for the ER and vesicle trafficking, TMV and TVCV differ in their requirements for the cytoskeleton in virus movement. The use of pharmacological agents has shown that TMV requires intact microfilaments for virus cell-to-cell movement, although there are disagreements about the role of microtubules in TMV intercellular spread (Harries et al., 2009b; Niehl and Heinlein, 2010; Schoelz et al., 2011). The TMV 126-kDa replicase forms cytoplasmic inclusions that associate with actin microfilaments and, thus, it has been proposed that TMV replication complexes that contain both MP^TMV^ and replicase traffic via the ER-actin network to PD for virus cell-to-cell spread (Harries et al., 2009b). In contrast to TMV, disrupting either the actin cytoskeleton or microtubules has no effect on TVCV cell-to-cell spread, nor does TVCV replicase associate with microfilaments. Thus, the cytoskeleton *per se* is not necessary for TVCV infection and spread (Harries et al., 2009b). In agreement with this, we have shown that the actin cytoskeleton is disrupted and becomes disorganized during TVCV infection, and MP^TVCV^ does not associate with microtubules (Levy et al., 2013). This further underscores the importance of the ER and endomembrane system in TVCV intracellular and intercellular trafficking. Indeed, a striking distinction between TMV and TVCV is that MP^TVCV^, in addition to localizing to ER membrane sites and PD, is also found in novel F-actin-containing nuclear filaments that associate with chromatin, and this nuclear function of MP^TVCV^ is necessary to promote efficient TVCV cell-to-cell movement (Levy et al., 2013).

### Begomoviruses

Like Tobamoviruses, the Begomoviruses (*Geminivirus* family) CaLCuV and SqLCV do not require CP to move cell to cell (Gardiner et al., 1988; Ingham et al., 1995; Pooma et al., 1996; Qin et al., 1998). However, in contrast to TMV and TVCV, CaLCuV and SqLCV encode a nuclear shuttle protein NSP in addition to the cell-to-cell movement protein (MP^CaLCuV^ or MP^SqLCV^), both of which are essential for virus intercellular transport and systemic infection (Lazarowitz and Beachy, 1999). This requirement for two proteins to traffic newly replicated progeny genomes to and through PD is the consequence of Begomoviruses having single strand DNA (ssDNA) genomes that are replicated in the nucleus and, thus, need to be efficiently transported across the nuclear envelope, as well as to and through PD (Pascal et al., 1994; Sanderfoot and Lazarowitz, 1995; Sanderfoot et al., 1996; Ward et al., 1997). Mutational analyses of CaLCuV or SqLCV MP and NSP, combined with transient expression studies in cultured insect cells or plant protoplasts, subcellular fractionation studies and electron microscopy lead to a model in which Begomovirus NSP and MP cooperatively act to move progeny viral genomes from their nuclear sites of replication to PD for intercellular transport (Pascal et al., 1994; Sanderfoot and Lazarowitz, 1995; Sanderfoot et al., 1996; Ward et al., 1997; Ward and Lazarowitz, 1999; Carvalho and Lazarowitz, 2004). According to this model, NSP binds progeny ssDNA genomes in the nucleus and transports them between the nucleus and cytoplasm. MP traps these NSP-genome complexes in the cytoplasm and redirects them to and through PD into neighboring cells, following which NSP is proposed to efficiently target the viral ssDNA genome into the nucleus to initiate new rounds of infection (Sanderfoot and Lazarowitz, 1996).

Despite these differences from Tobamoviruses in terms of replicating in a distinct compartment (the nucleus) and requiring an additional protein (NSP) to bind progeny genomes, MP^CaLCuV^ and MP^SqLCV^, like MP^TMV^ and MP^TVCV^, act to direct newly replicated viral genomes from their sites of replication to PD; and, also like TMV and TVCV, a variety of studies demonstrate the importance of the ER and endomembrane system in this process. MP^SqLCV^ co-fractionates with ER membranes in extracts from infected pumpkin plants and, based on immunolabeling studies, it localizes to ER-derived tubules that extend toward and cross the walls of SqLCV-infected cells at what may be immature PD (Ward et al., 1997). Fitting with this, MP^SqLCV^ and MP^CaLCuV^ each target to cortical regions of the ER when transiently expressed in insect cells and/or plant protoplasts (Sanderfoot and Lazarowitz, 1995, 1996; Ward et al., 1997; Carvalho and Lazarowitz, 2004). Mutational analyses show that this targeting to cortical ER is essential for virus infection and systemic spread; yet, both MP^SqLCV^ and MP^CaLCuV^ lack signal sequences and behave like peripheral membrane proteins (Sanderfoot and Lazarowitz, 1995; Sanderfoot et al., 1996; Carvalho and Lazarowitz, 2004; Lewis and Lazarowitz, 2010). Furthermore, as we demonstrated for MP^TMV^ and MP^TVCV^, SYTA regulates the ability of MP^CaLCuV^ to traffic to and modify PD, and CaLCuV cell-to-cell movement, via an endosomal recycling pathway that is distinct from the secretory pathway (Lewis and Lazarowitz, 2010). Hence, just like the Tobamoviruses TMV and TVCV, the ER and vesicle trafficking are necessary for MP^CaLCuV^ to traffic viral genomes to PD for cell-to-cell movement, and MP^CaLCuV^ does not appear to reach PD via the secretory pathway.

### Potyviruses

The Potyviruses have a single +RNA genome that is replicated in the cytoplasm. Thus, like the Tobamoviruses, moving simply the +RNA genome into new cells should, in principle, suffice to initiate infection. Nevertheless, studies mainly on *Tobacco etch virus* (TEV) and TuMV have shown that the mechanism employed by Potyviruses to replicate and target viral genomes to PD is more complex as it involves several viral-encoded proteins: CI; the coat protein CP; and the recently identified cell-to-cell movement protein PIPO (pretty interesting Potyvirus ORF), which is translated by ribosomal frameshifting within the Potyvirus polyprotein as P3N-PIPO (Dolja et al., 1995; Carrington et al., 1998; Wei et al., 2010a; Vijayapalani et al., 2012). CI is an RNA-binding protein with helicase and ATPase activities, and is essential for Potyvirus genome replication (Fernandez et al., 1995, 1998; Merits et al., 1998). CI also forms cylindrical inclusions (hence its name) in the cytoplasm adjacent to PD in infected cells, and conical structures that extend into PD (Rodriguez-Cerezo et al., 1997; Roberts et al., 1998; Sorel et al., 2014). Mutational analyses of TEV CP and CI (Dolja et al., 1994, 1995; Carrington et al., 1998); immunolabeling of CP and CI in cells infected with *Tobacco vein mottle virus* or *Pea Seed-borne mosaic virus* (Rodriguez-Cerezo et al., 1997; Roberts et al., 1998); and transient expression studies of fluorescently-tagged TuMV CI, CP and P3N-PIPO in *N. benthamiana* leaf cells (Wei et al., 2010a; Vijayapalani et al., 2012) lead to a model in which newly replicated viral CP-genome complexes move cell-to-cell and CI cooperatively interacts with P3N-PIPO, similar to NSP and MP in Begomovirus movement, to traffic these CP-genome complexes from viral replication sites to, and through, PD. According to this model, CI at perinuclear viral replication sites interacts with viral CP-genomes complexes, which may or may not be virions, and transports these to P3N-PIPO-modified PD. P3N-PIPO alters PD permeability and interacts with CI within inclusions to coordinate the formation of conical structures that are anchored within PD via P3N-PIPO, and through which viral CP-genome complexes are transported into adjacent cells (Sorel et al., 2014). In support of this model, Potyvirus CP is associated with the cylindrical inclusions, and viral CP and RNA have been localized to the CI conical structures that extend from and within PD (Rodriguez-Cerezo et al., 1997; Roberts et al., 1998; Sorel et al., 2014). CI also interacts with P3N-PIPO, and P3N-PIPO localizes to PD and affects PD gating (Wei et al., 2010a; Vijayapalani et al., 2012).

As for Tobamoviruses and Begomoviruses, a range of studies point to the importance of the ER and endomembrane system in the intracellular and intercellular trafficking of the Potyvirus genome. Potyvirus replication involves the sequential recruitment of ER-derived vesicles and the chloroplast outer membrane to create what has been described as viral replication factories supported by a globular structure in the perinuclear region of the cell (Wei and Wang, 2008; Wei et al., 2010b; Grangeon et al., 2012). In considering the coordination of virus replication and movement, and the multiple roles of CI, it is important to note that Potyviruses are in the Picornavirus superfamily and encode a single ORF, which is translated into a polyprotein that is proteolytically processed by three viral-encoded proteinases. The efficiencies of cleavage at the different processing sites vary, and precursors and their fully processed individual proteins can carry out different functions (Carrington et al., 1993; Lazarowitz, 2001). In particular, evidence supports the existence of CI-6K-VPg-NIa(Pro) and 6K-VPg-NIa(Pro) precursors, in which NIa(Pro) is the proteinase that processes these precursors, and the cleavage of CI from 6K-VPg-NIa(Pro) occurs slowly. The 6K-VPg-NIa(Pro) precursor targets the viral genome to initiate the replication process at ER sites, and CI would also be targeted to these sites through its linkage to 6K prior to cleavage (Carrington et al., 1993; Merits et al., 2002). The TEV and TuMV 6K protein (a.k.a. 6K2), which is an integral membrane protein, induces the proliferation of ER vesicles at ER exit sites that associate with *cis*-Golgi stacks (Restrepo-Hartwig and Carrington, 1994; Schaad et al., 1997; Wei and Wang, 2008). These 6K-associated ER-derived vesicles are proposed to then traffic to the outer chloroplast membrane, where they induce membrane invagination to form sites for potyvirus replication (Wei et al., 2010b). Transient expression studies of fluorescently-tagged TuMV 6K in *N. benthamiana* leaf cells and the localization of 6K in infected cells, the expression of compartment-specific markers and of dominant-negative mutants that disrupt COPI- or COPII-dependent vesicle trafficking, and the use of BFA, have lead to the suggestion that Potyviruses initiate translation of the viral genome on the ER and form 6K-vesicles at ER exit sites, and these vesicles then traffic via the Golgi to chloroplasts for virus replication. Thus, the early secretory pathway appears to be important for the formation of Potyvirus replication complexes (Wei and Wang, 2008; Wei et al., 2010b). In addition, 6K-YFP-labeled vesicles were observed to traffic along actin filaments, and this was inhibited by latrunculin B or a dominant-negative “tail” mutant of myosin XI-K. Thus, the actin cytoskeleton and myosin motors also appear to be required for 6K vesicles to traffic from the ER to chloroplast sites of virus replication (Wei et al., 2010b).

The ER and vesicle trafficking have also been implicated in CI-mediated transport of viral CP-genome complexes from these perinuclear replication sites to PD-associated inclusions, and in the targeting of P3N-PIPO to PD as well. Expression of a dominant-negative Sar1 mutant, or a high concentration of BFA, both inhibited the trafficking of fluorescently-tagged P3N-PIPO^TuMV^ to PD, and of tagged PDLP1, a plasmodesmal type I membrane receptor-like protein that traffics to PD via the secretory pathway (Amari et al., 2010; Wei et al., 2010a). However, in contrast to PDLP1, which was retained in the reticulate ER network in the presence of the Sar1 mutant, P3N-PIPO^TuMV^ was cytoplasmic when co-expressed with this Sar1 mutant (Wei et al., 2010a). This fits with the fact that P3N-PIPO is not predicted to contain a membrane-binding or membrane-spanning domain (Wei et al., 2010a; Vijayapalani et al., 2012), and suggests that although the ER and secretory pathway are necessary for P3N-PIPO^TuMV^ to target to PD, P3N-PIPO^TuMV^ itself does not traffic via the secretory pathway to reach PD. Thus, it appears that P3N-PIPO^TuMV^ may depend on a protein(s) that traffics through the secretory pathway in order to reach PD. Consistent with CI also not having predicted membrane-binding or membrane-spanning domains, and CI requiring P3N-PIPO^TuMV^ to localize to PD, neither BFA nor the SAR1 mutant inhibit CI self-association (aggregates) in the cytoplasm; but, both do inhibit CI localizing to PD when co-expressed with P3N-PIPO^TuMV^ (Wei et al., 2010a). Further underscoring the importance of the ER and vesicle trafficking in P3N-PIPO^TuMV^ targeting to PD, as well as the differences from PDLP1 trafficking, the actomyosin system does not appear to be required, at least for P3N-PIPO^TuMV^ to reach PD: latrunculin B or the expression of dominant-negative myosin XI-K or VIII-1 tail mutants did not inhibit P3N-PIPO^TuMV^ targeting to PD, although they did inhibit PDLP1 targeting to PD (Wei et al., 2010a).

As to the suggestion that P3N-PIPO^TuMV^ would interact with another protein(s) to reach PD, a recent study showed that P3N-PIPO^TuMV^ interacts *in planta* with Arabidopsis PCaP1, a hydrophilic cation-binding protein that stably associates with the plasma membrane via N-myristoylation (Vijayapalani et al., 2012). Although its function is unknown, PCaP1 interacts with Ca^2+^-bound calmodulin and phosphatidylinositol phosphates, and has been proposed to have a role in cell signaling (Nagasaki et al., 2008). BiFC studies show that P3N-PIPO^TuMV^ interacts with PCaP1 at the plasma membrane and in PD. Furthermore, TuMV cell-to-cell spread and systemic infection are severely inhibited in an Arabidopsis *pcap1* knockout line; thus, PCaP1 is necessary for TuMV infection (Vijayapalani et al., 2012). Based on these findings, it has been proposed that PCaP1 interacts with P3N-PIPO to potentially anchor the movement complex to the plasma membrane, from which PCaP1-bound P3N-PIPO can direct CI-bound viral RNPs (CP-genome complexes) to PD to move the viral RNPs into neighboring cells (Vijayapalani et al., 2012). The pathway by which CI-bound viral RNPs traffic to the cell periphery is still unknown, as is how PCaP1-bound P3N-PIPO would relocate from the plasma membrane to PD. In this regard, a recent study using pharmacological agents and dominant-negative mutants has implicated both the secretory pathway and the actomyosin system in TuMV cell-to-cell spread (Agbeci et al., 2013).

### Caulimoviruses

Based on their proposed modes of cell-to-cell movement, plant viruses have historically been characterized as “non-tubule forming,” which do not morphologically alter PD in a radical manner, or “tubule forming,” which drastically alter PD morphology by forming MP-containing large tubules that traverse PD and eliminate the desmotubule, which is the appressed ER at the PD core (Maule, 2008; Schoelz et al., 2011). The so-called tubule-forming viruses, as typified by CaMV, the Tospovirus *Tomato spotted wilt virus* (TSWV), and the Secovirus *Grape fanleaf virus* (GFLV), among others, move the viral genome as assembled icosahedral virions (CaMV, GFLV) or subviral particles (nucleocapsids and associated viral RNA-dependent RNA polymerase, e.g., TSWV). However, based on current molecular and cellular studies, we suggest that Tobamoviruses, Begomoviruses and Potyviruses can be viewed as representing a continuum of increasing complexity in virus movement. For TMV and TVCV, MP directly binds the viral genome to target it to PD and facilitate its intercellular movement. PD may appear to be swollen, but are not morphologically altered. In the case of SqLCV and CaLCuV, MP binds and directs NSP-ssDNA complexes, which are not virus particles, to and through PD, potentially guiding these complexes along tubules that may be modified desmotubule, although this latter point requires further investigation for viruses other than the phloem-limited SqLCV. For TEV and TuMV, CP-genome complexes, which may or may not be virions, are bound to CI, and MP (P3N-PIPO) targets CI to PD and enables CI to form cone-like extensions within PD, through which CP-genome complexes are proposed to move. PD are still recognizable as such, and MP is proposed to anchor the CI-containing cones to the PD. The state of the desmotubule is not clear; but, the fact that P3N-PIPO, like other viral MPs, can alter PD gating suggests that PD integrity is maintained. The tubule-forming viruses such as CaMV would represent the next and most extreme step in this progression: MP dramatically alters PD structure, including eliminating the desmotubule, to create large tubules that extend between cells and through which assembled virions or subviral particles tunnel into adjacent cells (Niehl and Heinlein, 2010; Schoelz et al., 2011). These differences, to a large extent, appear to reflect the different replication strategies of TVCV, CaLCuV, TuMV, and CaMV.

How the so-called tubule-forming MPs target to PD and form tubules is not understood, although the plasmodesmal integral membrane protein PDLP1 has been shown to have a role in the assembly of both MP^CaMV^ and MP^GFLV^ tubules at PD (Amari et al., 2010). Given this, it is not surprising that studies have implicated the endomembrane system in MP^CaMV^ and MP^GFLV^ tubule formation, although not in MP^CaMV^ associating with the plasma membrane when expressed in plant cell protoplasts (Huang et al., 2000; Carluccio et al., 2014). Transient expression of fluorescently-tagged MP^CaMV^ or other tubule-forming MPs in plant cell protoplasts is an established model for investigating the requirements for tubule formation. MP^CaMV^, although it does not have a transmembrane domain, accumulates at localized areas of the plasma membrane in protoplasts, from which tubules eventually extend outwards at the cell surface (Huang et al., 2000; Carluccio et al., 2014). In this model system, cytoskeletal assembly inhibitors do not affect tubule formation by MP^CaMV^ or MP^GFLV^, or the accumulation of MP^CaMV^ at localized areas of the plasma membrane; but, high concentrations of BFA that disrupt the endomembrane system do inhibit tubule formation (Huang et al., 2000; Laporte et al., 2003).

Interactions of MP^CaMV^ with pectin methylesterase (Chen et al., 2000) and PRA1, a Rab GTPase receptor that localizes to prevacuolar compartments (PVCs) (Huang et al., 2000), have been cited as further implicating the endomembrane system in tubule formation, and a recent study provides evidence for MP^CaMV^ trafficking in the endocytic pathway (Carluccio et al., 2014). In this study, fluorescently-tagged MP^CaMV^, when transiently expressed in protoplasts, was found on endosomal vesicles at ~10 h post transfection, shortly after MP^CaMV^ was observed to accumulate at localized areas of the plasma membrane and tubule elongation from the cell surface began (~6–8 h post transfection). Labeling with the lipophilic dye FM4-64 suggested that a subset of the MP^CaMV^-labeled vesicles are derived from the plasma membrane (Carluccio et al., 2014). The authors identified three conserved YXXΦ Tyr-sorting motifs in MP^CaMV^. Such motifs are recognized by the μ-subunit of adaptor protein (AP) complexes for cargo selection on clathrin-coated vesicles, and have previously been identified in MP^GFLV^ and in one of the so-called triple gene block movement proteins encoded by the Pomovirus *Potato mop-top virus* (Laporte et al., 2003; Haupt et al., 2005). MP^CaMV^ YXXΦ motifs appeared to be essential for infectivity and, consistent with endocytic trafficking being important for infection, CaMV did not infect *ben1*(*min7*) and *ben2*(*vps45*) plants, two Arabidopsis mutant lines with defects in the trafficking of early endosomes through the TGN/EE, a dynamic tubular-vesicular trans-Golgi network in plant cells that is the main sorting hub for secretory and vacuolar traffic, as well as the destination for endocytic cargo (Zeenko et al., 2002). Based on mutational studies, at least one YXXΦ motif is needed for MP^CaMV^ to form tubules at the protoplast surface, to target to PD in leaf epidermal cells, and to interact *in vitro* with the μ1 adaptin subunit of Arabidopsis AP-1, which is mainly at the trans-Golgi; but, MP^CaMV^ lacking all three YXXΦ motifs still interacts with PDLP1 *in vitro*. Interestingly, the MP^CaMV^ triple YXXΦ mutant still appeared to associate with the plasma membrane at 8 h post transfection, but did not subsequently localize to endosomes; rather, it appeared in cytoplasmic aggregates, at this and later times post-transfection, that did not label with FM4-64 (Carluccio et al., 2014). Further suggesting a role for endocytic trafficking in MP^CaMV^ function, blocking AP-2-mediated endocytosis with tyrphostin A23 caused MP^CaMV^ to remain at the plasma membrane of protoplasts in a diffuse pattern and not appear on endosomes. In addition, a high concentration of BFA caused MP^CaMV^ to accumulate in large FM4-64 labeled internal patches, and a subset of MP^CaMV^-marked endosomes co-localized with RabF2b GTPase (ARA7), a marker for MVBs/PVCs (Carluccio et al., 2014). Based on these findings, MP^CaMV^ was proposed to reach the plasma membrane by an unknown mechanism, following which it traffics via an endocytic recapture pathway to reach PD and interact with PDLP1 to form tubules for virus intercellular movement. It was also proposed that excess MP^CaMV^ is targeted for degradation via MVBs (Carluccio et al., 2014). Verification of this model will require more direct studies and further characterization of endosomal trafficking pathways in plant cells, which are complex and, at times, controversial (Chen et al., 2000; Ryabova et al., 2002, 2004; Ueki and Citovsky, 2005; Craxton, 2007; Thiebeauld et al., 2009). Nevertheless, our findings show that whatever endocytic route MP^CaMV^ may use to reach PD, this is distinct from the SYTA-regulated recapture pathway that our studies have identified (Lewis and Lazarowitz, 2010) (Levy, Zheng and SGL, submitted).

### A working model for SYTA action in virus movement

As underscored by these four and other proposed schemes for virus movement, progress has been made in identifying viral proteins required for movement and their interacting partners, but we still do not understand the mechanisms by which MPs reach PD and alter their permeability or the biophysics of moving viral genome-protein complexes. Our goal here was to examine whether SYTA function might be a general requirement for plant virus cell-to-cell movement. We found that SYTA acts as a common mechanism to regulate plant virus the cell-to-cell movement, but not a universal one. Synaptotagmins are important for membrane trafficking in eukaryotic cells, acting to regulate vesicle fusion at the plasma membrane for exocytosis and endocytosis (Chapman, 2008; Moghadam and Jackson, 2013). Consistent with the established roles of synaptotagmins in animal cells, SYTA localizes to the plasma membrane in plant cells (Schapire et al., 2008; Lewis and Lazarowitz, 2010), and our studies have shown that SYTA regulates endocytosis and an endocytic recycling pathway to the plasma membrane (Lewis and Lazarowitz, 2010). Importantly, SYTA directly interacts with MP^TMV^ and MP^TVCV^, and with MP^CaLCuV^ and MP^SqLCV^, and our functional studies show that the dominant-negative mutant SYTA^ΔC2B^, which is defective in mediating endocytosis, must be at the plasma membrane in order to interfere with MP^TMV^ and MP^CaLCuV^ cell-to-cell trafficking (Lewis and Lazarowitz, 2010; A. Levy, J. Zheng, and SGL, submitted). Hence, the SYTA-regulated recycling pathway at the plasma membrane is necessary for these distinct Tobamovirus and Begomovirus MPs to alter PD gating and transport virus genomes through PD. This leads us to propose that SYTA acts to target viral MPs and their genome cargos to PD via this endocytic recapture pathway. This model does not preclude an additional role for SYTA in the mechanism by which MPs alter PD permeability. Without an available *syta* null mutant, we cannot conclude whether SYTA is both necessary and sufficient for MP^CaLCuV^, MP^TVCV^, and P3N-PIPO^TuMV^ to reach and alter PD or other Arabidopsis SYTs may contribute in an overlapping manner to this process. Our characterization of the remaining four Arabidopsis SYTs shows that *SYTA, SYTB* and *SYTC* each have distinct patterns of expression, and that neither SYTB nor SYTC appears to mediate plant virus cell-to-cell movement via PD (H. Shimada-Beltran and SGL, unpublished; A. Uchiyama, J. Zheng, P. Javia, and SGL, in preparation). Our studies further show that *SYTE*, like *SYTA*, is highly expressed in all cell types and at all stages of development in Arabidopsis. Thus, it remains possible that SYTE could have an overlapping function with SYTA in regulating virus intercellular movement. Additional studies are required to clarify this point.

How can our finding that SYTA is needed for TVCV, CaLCuV, and TuMV MP function and cell-to-cell movement, but not for CaMV infectivity and spread, inform our thinking? SYTA regulates the cell-to-cell trafficking of MP^TVCV^ and P3N-PIPO^TuMV^ via PD (Figure 3, Table 3), as well as that of MP^CaLCuV^ and MP^TMV^ (Lewis and Lazarowitz, 2010). P3N-PIPO^TuMV^ interacts with PCaP1, and this interaction is proposed to anchor P3N-PIPO^TuMV^ to the plasma membrane, from which PCaP1-bound P3N-PIPO^TuMV^ with its cargo (CI-bound viral RNPs) is directed to PD. SYTA directly interacts with MP^CaLCuV^, MP^TMV^ and MP^TVCV^ (Lewis and Lazarowitz, 2010; Levy, Zheng, and SGL, submitted), but we do not know whether it interacts with P3N-PIPO^TuMV^. However, PCaP1 is not required for Tobamovirus infection: *Oilseed rape mosaic virus*, which like TVCV is a subgroup 3 Tobamovirus, is equally infectious on an Arabidopsis *pcap1* knockout line and wild type Col-0 plants (Vijayapalani et al., 2012). Hence, the common link here is SYTA. P3N-PIPO^TuMV^ requires the secretory pathway to reach PD, although it does not itself traffic via the secretory pathway to PD (Wei et al., 2010a). PCaP1 is a hydrophilic protein that stably associates with the plasma membrane via N-myristoylation (Vijayapalani et al., 2012) and, thus, it would not reach the plasma membrane via the secretory pathway. However, SYTA does traffic to the plasma membrane via the secretory pathway (Lewis and Lazarowitz, 2010). Therefore, fitting with our model for SYTA targeting MP^TMV^ and MP^CaLCuV^ and their genome cargos to PD, we propose that P3N-PIPO^TuMV^ binds to PCaP1 at the plasma membrane, following which the SYTA-regulated recycling pathway directs this complex, with its cargo, to PD.

MP^CaMV^ is proposed to reach the plasma membrane by an unknown mechanism and then traffic via an endocytic recapture pathway to reach PD, where it interacts with PDLP1 to form tubules for virus intercellular movement (Amari et al., 2010; Carluccio et al., 2014). Our results (Figure 2B, Table 4) suggest that if MP^CaMV^ uses an endosomal recycling pathway to traffic from the plasma membrane to PD, this pathway is distinct from the SYTA-regulated recycling pathway. The key distinctive features of MP^CaMV^ and CaMV movement—compared to TVCV, CaLCuV, and TuMV—are that MP^CaMV^ assembles into large tubules that dramatically alter PD by eliminating the desmotubule; MP^CaMV^ interacts with the PD transmembrane protein PDLP1, which is important for tubule assembly within PD; and MP^CaMV^ tubules dramatically widen what was the PD channel so that CaMV virions can traffic cell to cell, but it does not direct these virus particles, and hence the viral genome, to PD (Harries et al., 2009a; Schoelz et al., 2011). Given the large size of CaMV icosahedral virus particles, it seems that a specialized machinery and cytoskeletal-guided movement, rather than a vesicle trafficking pathway, would be better suited to transporting the encapsidated genome to PD. Indeed, the CaMV P6 protein interacts with both viral CP and MP^CaMV^ (Himmelbach et al., 1996; Hapiak et al., 2008), and the available evidence suggests that aggregates or inclusions of P6 may transport CaMV virions along microfilaments to the assembled MP^CaMV^ tubules, where viral-encoded VAP, interacting with CP and MP^CaMV^, mediates guidance of virions through the tubules and into neighboring cells (Stavolone et al., 2005; Harries et al., 2009a). Neither the endomembrane system or an intact cytoskeleton appear to be required for MP^CaMV^ to reach the plasma membrane, at least in protoplasts, and MP^CaMV^ appears to be recruited onto endosomes through an interaction of its Tyr-sorting motifs with AP-2 at the plasma membrane (Huang et al., 2000; Carluccio et al., 2014). Thus, it seems that SYTA is not involved in MP^CaMV^ reaching the plasma membrane, nor would an interaction with SYTA be needed to direct MP^CaMV^ onto endosomes, although, in principle, SYTA could mediate the fusion at the plasma membrane to create these endosomes. However, all of our data indicate that the important step is the SYTA-regulated endosomal recycling pathway directing MP-containing complexes from the plasma membrane *to* the PD channel for genome transport into neighboring cells. Thus, since MP^CaMV^ interacts with a PD transmembrane protein (PDLP1) and becomes a physical component of the channel, it may be that MP^CaMV^ trafficking to PD involves pathways that are independent from the SYTA-regulated endosomal recapture pathway. It is possible that MP^CaMV^ tubules, by so drastically altering the channel, including removing the desmotubule, render the channel unrecognizable as a “PD,” and hence the SYTA-regulated endosomal recycling pathway does not traffic macromolecular complexes to these sites. A corollary of this is that recognition of the appropriate vesicle docking site(s) at PD may require an intact desmotubule and/or its continuity with the cortical ER. Finally, an intriguing, and not mutually exclusive, possibility is that beyond simply delivering MPs and their cargos to PD, the interaction with SYTA may also be involved in MP activity to alter PD permeability. Our current studies are exploring these possibilities.

## Author contributions

Asako Uchiyama, Harumi Shimada-Beltran, Amit Levy, Judy Y. Zheng and Sondra G. Lazarowitz designed research and analyzed data. Asako Uchiyama performed the TVCV and CaMV infectivity assays, and all of the movement assays, and statistical analyses of the data. For the P3N-PIPO^TuMV^ movement assays, Asako Uchiyama worked with and supervised Parth A. Javia. Harumi Shimada-Beltran and Judy Y. Zheng carried out TuMV infectivity assays and statistical analyses of the data. Judy Y. Zheng was supervised by Asako Uchiyama and Sondra G. Lazarowitz. Amit Levy performed statistical analyses of P3N-PIPO^TuMV^ movement assays and sqPCR analyses of CaMV DNA in systemic leaf extracts. Sondra G. Lazarowitz supervised all aspects of this work. Amit Levy and Sondra G. Lazarowitz made the figures and tables for this manuscript. Sondra G. Lazarowitz wrote the manuscript with contributions from Amit Levy and Harumi Shimada-Beltran.

### Conflict of interest statement

The authors declare that the research was conducted in the absence of any commercial or financial relationships that could be construed as a potential conflict of interest.

## Footnotes

^1^Hereafter, MP refers to the virus cell-to-cell movement protein that gates PD, with the virus name in superscript. Virus-specific names denote additional movement proteins involved in intracellular transport.

^2^By convention, the nomenclature for animal and yeast proteins capitalizes only the first letter, whereas in the plant nomenclature all 3 letters are capitalized. We will use the plant convention here, with the species specifically designated.
